# Predictability of a dental implant prognosis system: A retrospective study

**DOI:** 10.1002/jper.70110

**Published:** 2026-03-06

**Authors:** Kristen Young, Michelle Tong, Yu‐Ting Yeh, Richard T. Kao, Guo‐Hao Lin

**Affiliations:** ^1^ Division of Periodontology, Department of Orofacial Sciences, School of Dentistry University of California San Francisco San Francisco California USA; ^2^ Department of Periodontics & Oral Medicine, School of Dentistry University of Michigan Ann Arbor Michigan USA; ^3^ Department of Dentistry, School of Dentistry National Yang Ming Chiao Tung University Taipei Taiwan

**Keywords:** dental implants, dentistry, diagnosis, prognosis, risk factor(s)

## Abstract

**Background:**

This study aimed to evaluate the predictability of the dental implant prognosis system proposed by Kwok et al. in 2023 by assessing implant survival rates across different initial prognosis categories over a 5‐year period.

**Methods:**

Data were retrospectively collected from patients who received at least one dental implant from 2013–2022 at a single university‐affiliated dental center. Eligible participants received baseline examinations at the time of implant‐supported prosthesis delivery, with follow‐up examinations at least 12 months after. Implants were categorized into favorable, questionable, unfavorable, and hopeless, based upon the prognosis categories proposed by Kwok et al. in 2023. Recorded measurements included: age, sex, follow‐up duration, initial and latest implant prognoses, supportive care, history of grade C periodontitis, smoking, diabetes, and the use of intravenous bone sparing agents. Descriptive analysis was employed for data interpretation.

**Results:**

This study included 651 implants placed in 291 patients. Implants initially classified as having a favorable prognosis exhibited excellent long‐term survivability, while those deemed unfavorable at baseline showed the greatest likelihood of deteriorating to a hopeless prognosis. At the latest follow‐up, survival rates for implants initially categorized as favorable, questionable, and unfavorable were 100%, 93.5%, and 33.3%, respectively. Cox regression analysis identified initial prognosis, uncontrolled diabetes, and a history of grade C periodontitis as statistically significant risk factors for implant failure, with hazard ratios of 9.70, 35.14, and 66.48, respectively.

**Conclusions:**

The dental implant prognosis system proposed by Kwok et al. is a reliable tool in assessing implant survivability within 5 years. Future research should investigate its long‐term performance and the relationship between various influencing factors and implant prognosis.

## Plain language summary

This study explored how well a new system developed by Kwok and colleagues in 2023 can predict the long‐term success of dental implants. We reviewed the records of 291 patients who received a total of 651 implants at a university‐affiliated dental center between 2013 and 2022. Each implant was classified into one of four categories—favorable, questionable, unfavorable, or hopeless—at the time the prosthesis was delivered, and patients were followed for up to 5 years. The findings showed clear differences in survival between the groups. Implants with a favorable prognosis had excellent outcomes, with all surviving after 5 years. Those with a questionable prognosis also performed well, with 93.5% still in place. By contrast, only one in three implants in the unfavorable group survived to the 5‐year mark. Additional analysis showed that the initial prognosis, along with uncontrolled diabetes and a history of severe gum disease, were strong risk factors for implant failure. These results suggest that the proposed prognosis system is a valuable tool to help dentists and patients understand the likelihood of implant survival over time. More research is needed to confirm how well it works in the longer term.

## 1 INTRODUCTION

Prognosis is a term for predicting a disease's progress, course, and outcome. Several prognosis systems have been proposed for natural dentition. Hirschfeld and Wasserman developed the initial prognosis system for questionable teeth in 1978.^1^ Based on the response to therapy and number of teeth lost, the authors divided tooth loss into three different categories: well‐maintained, downhill, and extreme downhill. The authors observed different patterns of questionable teeth lost in each group; with the least number of teeth lost in the well‐maintained group and the most significant number in the extreme downhill group. In 1984, Becker et al. devised a prognosis system derived from clinical and radiographic findings, categorizing teeth into three groups: good, questionable, and hopeless based on specific criteria. Over a 5‐year period, the authors observed an annual tooth loss rate of 0.36 teeth for untreated patients, 0.22 for treated but not maintained, and 0.11 for those who received both treatment and maintenance.^2^ To develop a more accurate and comprehensive prognosis system, McGuire proposed a prognosis system in 1991 that considered both individual teeth and overall prognosis. McGuire's classification, based on tooth loss, encompassed the categories of good, fair, poor, questionable, and hopeless prognosis.^3^

While there are several prognosis systems proposed for natural dentition^1^, ^2^, ^3^, ^4^, systems for determining implant prognosis have not been as well developed. Since Branemark introduced osseointegrated dental implants in 1969, the use of dental implants as replacements has gained popularity due to their functionality, esthetics and more^5^. The prevalence of dental implants increased on average by 14% per year from 1999 to 2000 and 2015 to 2016.^6^ With the increasing placement of dental implants, peri‐implant diseases and implant failure have gradually emerged as possible complications.^7^ Therefore, determining the prognosis for dental implants is critical. It will guide care and provide us with a better understanding of how risk factors can influence their treatment course.

In 2015, Decker et al. introduced a prognosis system to evaluate individual implants and to advise on treatment modalities for peri‐mucositis and varying stages of peri‐implantitis. Specifically, the system evaluated the probability of achieving implant stability after using nonsurgical and/or surgical interventions. The prognosis scale comprises of favorable, questionable, unfavorable, and hopeless. A favorable implant prognosis is associated with peri‐implant mucositis or early stages of peri‐implantitis, where treatment recommendation often includes nonsurgical therapy and the use of local/ systemic antibiotics. A questionable prognosis is associated with implant placement in a previously dentate site with a history of moderate periodontitis. Furthermore, an unfavorable or hopeless implant prognosis is related to a site that has had advanced periodontitis dentition.^8^ Despite their full effort, the authors emphasized their prognosis system relies heavily on the amount of bone loss relative to the implant length as an indicator of disease severity. Thus, if an implant was not placed at an ideal position, a downgrade of a prognosis category is expected.

To address this concern, Kwok et al.^9^ introduced an implant prognosis system focusing on peri‐implant supporting tissue stability rather than predicting the result of implant treatment with advanced bone loss.^8^ According to this newly developed system, implant stability is dependent on plaque control, implant position and/or restoration design, a history of periodontitis, supportive care, smoking, diabetes mellitus, osteoporosis, and other elements including the use of bone sparing agents (i.e., bisphosphonate), radiation therapy, occlusion, and lastly, genetic phenotypes. Much like the prognosis system applied to natural teeth^4^, the proposed implant prognosis system by Kwok et al. comprises four categories: favorable, questionable, unfavorable, and hopeless. An implant with a favorable prognosis will likely maintain tissue stability with appropriate supportive therapy. While indications of peri‐mucositis may be observed, there should be the absence of peri‐implantitis with no notable local/systemic influencing factors. A questionable prognosis indicates that tissue stability may or may not be maintained when signs of peri‐implantitis are present, or when the patient exhibits a moderate level of local and/or systemic risk factors. However, the prognosis may improve with appropriate treatment and/or positive modifications to the contributing factors. Unfavorable is characterized by signs of peri‐implantitis and presence of significant local/systemic risk factors. Implant tissue stability is unlikely to be maintained and no improvement in tissue status and local/systemic factors. An implant is deemed hopeless when osseointegration is not attained or is lost due to peri‐implantitis, or if it is placed at an angulation or position that renders it unusable (Supplemental Table 1 in the online *Journal of Periodontology*).^9^

Having a reliable prognosis system is essential to aid clinicians in assessing the short‐ and long‐term stability of an implant. For clinicians, an accurate prognosis can have a significant influence on the choice of treatment and overall care planning. Kwok et al.’s implant prognosis system^9^ shows great potential to effectively predict implant survivability. While promising, this system has not yet been validated in a clinical setting. Therefore, this study seeks to evaluate the predictability of the dental implant prognosis system proposed by Kwok et al. in 2023 by assessing implant survival rates across different initial prognosis categories over a 5‐year period.

## 2 MATERIALS AND METHODS

### 2.1 Study design

Electronic dental records from patients who received at least one dental implant at the University of California, San Francisco (UCSF) School of Dentistry Postgraduate Periodontics Clinic and Periodontics Faculty Clinic between January 2013 and December 2022 were reviewed. Records were reviewed for patients who had two separate recorded entries with baseline examination at the time of implant‐supported prosthesis delivery and subsequent follow‐up spaced at least 12 months after. Charts were included in this study when the presence of radiographs was available on both examinations. Charts, however, were excluded from the study if insufficient information was present to properly determine prognosis, including unknown supportive care protocol and history. If an implant was present at the time of prosthesis delivery and was absent at the time of the latest examination, the implant failure was recorded, and the final implant prognosis was categorized as hopeless. In contrast, implants that failed prior to prosthesis delivery were excluded from this study. The implant prognosis categories were assigned by two independent examiners (K.Y. and M.T.) and verified by a board‐certified faculty periodontist (G.H.L.). The kappa statistic was used to evaluate the inter‐examiner reliability between the two examiners (K.Y. and M.T.) in assessing implant prognosis based on identified influencing factors and radiographic evaluations of 20 representative patients with implants independently.

The following patient data were collected: age, sex, length of follow‐up period, initial and latest implant prognosis, and potential influencing factors were recorded. Diabetes mellitus was categorized as non‐diabetic, controlled (HbA1c < 7%), or uncontrolled (HbA1c ≥ 7%). Frequency of supportive care was divided into once per year or twice or more per year.^10^ Data, including smoking status, history of grade C periodontitis, and use of intravenous (IV) bone sparing agents, were also recorded. Based on radiographs and the recorded influencing factors, implant prognosis was assigned based on categories proposed by Kwok et al.,^9^ favorable, questionable, unfavorable, or hopeless. The implant prognosis at the Initial and latest examination was then compared and documented.

### 2.2 Statistical analysis

After data acquisition, descriptive analysis was conducted to evaluate the dataset. Baseline demographic characteristics and potential influencing factor exposures were summarized using the mean and standard deviation for continuous variables, and frequencies and percentages for categorical variables. Univariate Cox proportional hazards models were used to investigate associations between influencing factors and implant loss using the individual implant as the analysis unit. Hazard ratio (HR) with 95% confidence interval (CI) was calculated. The influence of the Initial implant prognosis on implant loss was also analyzed using Cox regression. A *p* value < 0.05 was deemed statistically significant. A Kaplan–Meier curve was employed to depict the distribution of time to failure across the levels of initial prognosis. Additionally, a heat map was generated to depict implant survivability analysis based on the position of the implant location for those initially categorized with a favorable prognosis.

Patient confidentiality was protected in compliance with the Privacy regulations of the Federal Health Insurance Portability and Accountability Act of 1996 (HIPAA). Additionally, the study protocol underwent proper review and was approved by the UCSF Institutional Review Board (ID number: 24–41437). The Strengthening the Reporting of Observational Studies in Epidemiology (STROBE) guidelines were utilized to ensure comprehensive reporting.^11^

## 3 RESULTS

After reviewing 861 electronic dental records, 651 implants from 291 patients satisfied the inclusion criteria and were subsequently incorporated into this study. Of these patients, 51.5% were male and 48.5% were female, with a mean age of 67.5 ± 17.7 years (range of 25 to 91). Table 1 presents the baseline demographic characteristics and potential influencing factor exposures among the study participants. The inter‐examiner reliability for implant prognosis assessment demonstrated excellent agreement, with a kappa value of 0.90. Any discrepancies were subsequently reviewed and resolved with the input of a third examiner (G.H.L.).

**TABLE 1:** Baseline demographic characteristics and potential influencing factor exposures among the study participants.

Parameter	Total (*N* = 651)		Favorable prognosis (*N* = 538)		Questionable prognosis (*N* = 107)		Unfavorable prognosis (*N* = 6)	
Mean age (standard deviation)	68	(12.4)	67.7	(12.7)	69.6	(10.8)	66.3	(8.3)
Characteristic	*N*	(%)	*N*	(%)	*N*	(%)	*N*	(%)
Sex								
Female	298	(45.8)	254	(47.2)	43	(40.2)	1	(16.7)
Male	353	(54.2)	284	(52.8)	64	(59.8)	5	(83.3)
Supportive care								
1 cleaning/year	305	(46.9)	274	(50.9)	30	(28.0)	1	(16.7)
≥2 cleanings/year	346	(53.1)	264	(49.1)	77	(72.0)	5	(83.3)
Diabetes								
Controlled	586	(90.0)	538	(100.0)	48	(44.9)	0	
Uncontrolled	65	(10.0)	0		59	(55.1)	6	(100.0)
Smoking								
Never	388	(59.6)	342	(63.6)	46	(43.0)	0	
Former	224	(34.4)	186	(34.6)	32	(29.9)	6	(100.0)
Current	39	(6.0)	10	(1.9)	29	(27.1)	0	
IV bone metabolic agent								
Never	650	(99.8)	537	(99.8)	107	(100.0)	6	(100.0)
Past use	1	(0.2)	1	(0.2)	0		0	
History of periodontal disease								
Non‐grade C history	576	(88.5)	538	(100.0)	38	(35.5)	0	
Grade C history	75	(11.5)	0		69	(64.5)	6	(100.0)

Abbreviation: IV, intravenous.

**FIGURE 1:**
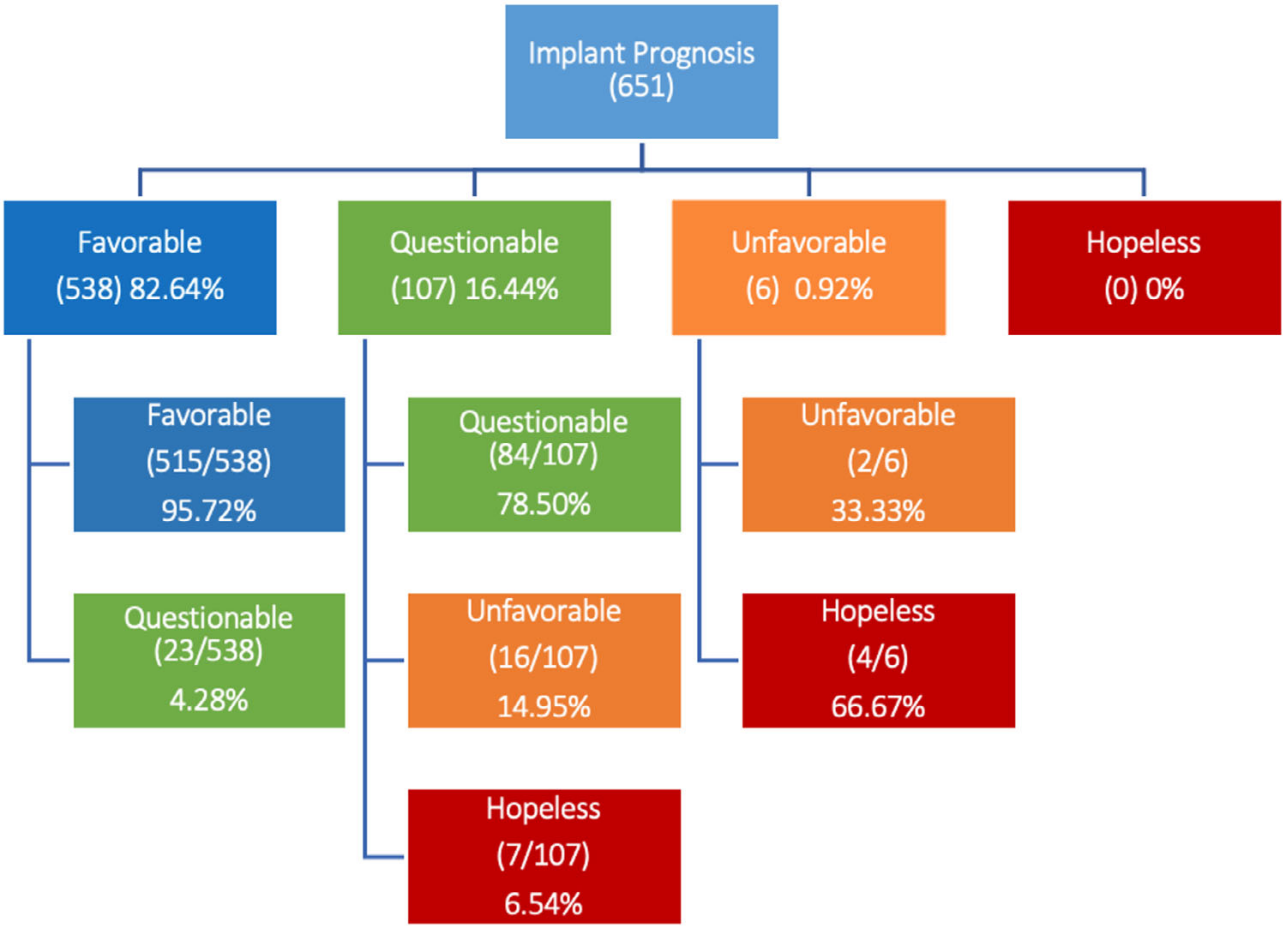
The initial prognosis of the included implants and their survival outcomes at the latest oral examination.

Upon initial examination, 538 implants were categorized as favorable, 107 were questionable, six were unfavorable, and none were hopeless (Figure 1). At the latest examination, 11 implants were categorized as hopeless and consequently extracted. Of the failed implants, four had an initial unfavorable prognosis, and seven had an initial questionable prognosis. It should be noted that 10 individuals classified as current smokers had their implants initially assigned a favorable prognosis, as they were nonsmokers at the time of implant placement but resumed smoking during the follow‐up period.

The observation period ranged from 12 to 132 months, with an average of 34 ± 21.53 months between the initial and latest examination. When comparing baseline and final prognosis, implants that were initially given a favorable prognosis, 515 (95.7%) maintained a favorable prognosis, and 23 (4.3%) were downgraded to a questionable prognosis; none were changed to an unfavorable or hopeless prognosis at the latest examination. Of the implants initially categorized as questionable, 84 (78.5%) remained with a questionable prognosis, 16 (15%) were reclassified to an unfavorable prognosis, and seven (6.5%) were deemed hopeless. For implants initially categorized as unfavorable, 2 (33.3%) remained unchanged, and four (67%) were reclassified to hopeless. No implants were categorized as hopeless at baseline.

Among implants classified as having a hopeless prognosis, outcomes were frequently associated with the presence of influencing factors. Implant failures occurred in two patients whose implants were placed during periods of poor glycemic control (HbA1c 9.8% and 9.9%). The remaining implants in these patients exhibited notable radiographic bone loss consistent with peri‐implantitis and were categorized as having an unfavorable prognosis. Despite patients achieving improved glycemic control over time, ideally controlled glycemic levels were not observed at any point during follow‐up. In fact, two patients initially presenting with controlled diabetes experienced worsening metabolic status, with one reaching an HbA1c of 10.3%. Another failed implant occurred in a patient who resumed smoking after implant placement, while seven additional implants in this individual were identified as questionable but showed no signs of peri‐implantitis. Finally, a single patient with no identifiable influencing factors experienced a singular, unexplained implant loss despite regular maintenance. Collectively, the data from these implant outcomes illustrate the multifactorial nature of implant placement failure and that, despite our best efforts with regular supportive care, implants can and do occasionally fail without an identifiable cause.

As shown in the Cox regression analysis (Table 2), implants initially classified with an unfavorable prognosis were significantly more likely to fail compared to those deemed questionable (HR = 9.70, 95% CI [2.76, 34.71], *p* < 0.001). Patients with uncontrolled diabetes exhibited a markedly higher risk of implant loss (HR = 35.14, 95% CI [7.58, 162.85], *p* < 0.001). Additionally, individuals with a history of grade C periodontitis demonstrated a substantially elevated risk of implant failure (HR = 66.48, 95% CI [8.50, 519.67], *p* < 0.001). In contrast, no statistically significant association was observed between the frequency of supportive care visits (≥ 2 times/year vs. once/year) and implant failure (HR = 7.01, 95% CI [0.90, 54.92], *p* = 0.06). Notably, the potential impact of bone sparing agents could not be assessed due to the minimal number of patients using them (*n* = 1).

**TABLE 2:** Assessment of relative hazard of implant loss across various influencing factor categories using Cox regression analysis.

Influencing factors	Hazard ratio	*p‐*value	95% confidence interval
Initial prognosis			
Questionable	1		
Unfavorable	9.7	< 0.001^*^	2.76–34.71
Supportive care			
1 cleaning/year	1		
≥2 cleanings/year	7.01	0.06	0.90–54.92
Diabetes			
Controlled	1		
Uncontrolled	35.14	< 0.001^*^	7.58–162.85
Smoking			
Never	1		
Former	0.95	0.94	0.27–3.37
Current	1.31	0.80	0.16–10.91
History of periodontal disease			
Non‐grade C periodontitis	1		
Grade C periodontitis	66.48	< 0.001^*^	8.50–519.67

* Indicates statistical significance at *p *< 0.05.

**FIGURE 2:**
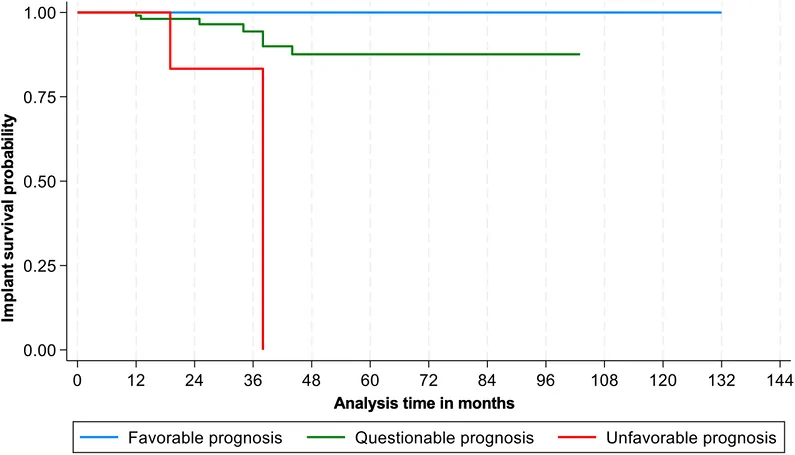
An unadjusted Kaplan–Meier survival plot depicting the decline in implant survivability over time in those initially with questionable and unfavorable prognoses.

A Kaplan–Meier survival plot (Figure 2) assessed implant survival over time based on the initial assigned prognosis. Implant prognosis categories were monitored, and the percentage of implant survival over time was mapped. Over the observed interval of 132 months, implants with an initial favorable prognosis all had desirable outcomes. In contrast, those with an initially unfavorable prognosis had the highest risk of progressing to a hopeless prognosis and subsequent implant failure. Implants with initially questionable prognoses mainly remained consistent with negligible decline in implant retention over the study period. Implants initially regarded as unfavorable had a higher tendency of poor stability, with a noticeable drop in implant survivability at approximately 20 months and a substantial decline in survivability around the 40‐month mark. Overall, implants in the initially favorable and questionable groups had the most consistent stability throughout the study period.

**FIGURE 3:**
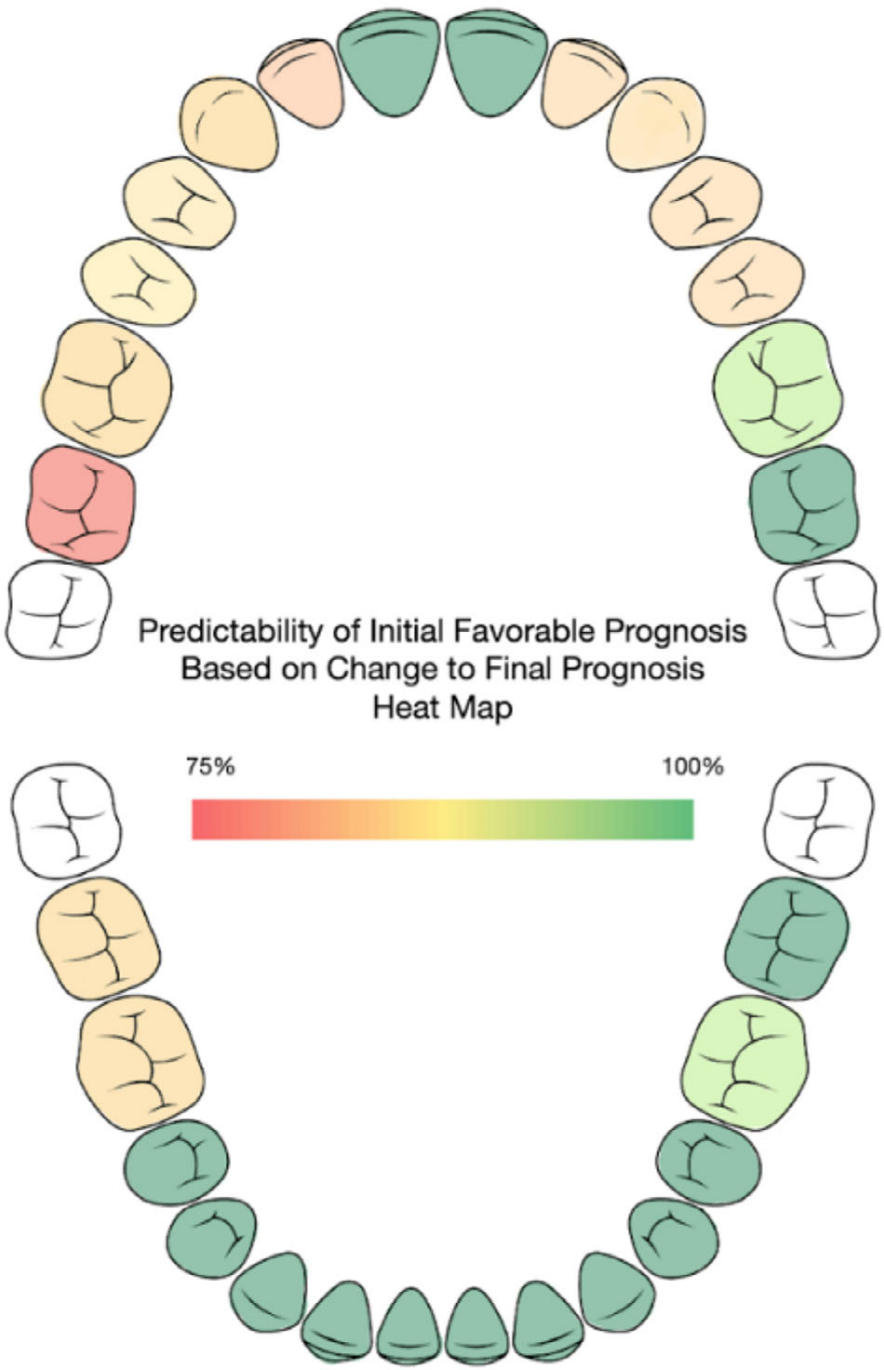
A heat map illustrating implant survivability analysis based on the anatomical position of the implant location. Here, the figure indicates that, in general, for implants initially given a favorable prognosis, mandibular implants are more predictable than maxillary implants.

A heat map (Figure 3) was used to correlate implant survivability with implant site in the mouth. The heat map demonstrates the predictability of an initial favorable prognosis for each tooth replacement site in the mouth. Based on the heat map, the initial prognosis assigned to predict the survivability of maxillary teeth was not as predictable as the initial prognosis assigned to replace mandibular teeth. For mandibular replacements, prognoses of anterior and premolar sites were more reliable compared to those of molar positions. Regarding maxillary replacements, the initial prognosis was only consistently predictive of implant outcome for the central incisors.

## 4 DISCUSSION

Over the years, several periodontal prognosis systems have been implemented^1^, ^2^, ^3^, ^4^, and only some of their accuracies were evaluated.^3^, ^4^, ^12^ McGuire's prognosis classification^3^ was evaluated and reported to be 81% accurate at follow‐ups within 5–8 years but later declined to approximately 50% when the teeth categorized in good prognosis were excluded.^4^ The accuracy of Kwok and Caton's periodontal prognosis classification^4^ was also validated and found that the tooth survival rate at the latest examination for those with an initial favorable, questionable, unfavorable, and hopeless prognosis was 97.9%, 90.7%, 62.5%, and 17.7%, respectively, within a 5‐year period.^12^ Such validations are essential to ensure the reliability and clinical applicability of systems used to guide treatment planning. This study builds on prior efforts to validate periodontal prognosis models by applying a similar framework to assessing the implant prognosis proposed by Kwok et al. ^9^

The result of our study indicated that the implant prognostic system proposed by Kwok et al. 2023^9^ displayed accurate predictive capacity in estimating implant survivability. At the latest examination, implant survival rates for those with initially favorable, questionable, and unfavorable conditions were 100%, 93.5%, and 33.3%, respectively. Additionally, implants with an initial prognosis of favorable, questionable, and unfavorable that remained unchanged in the same category were 95.7, 78.5, and 33.3, respectively. Strikingly, no implant failures were observed in the initially favorable group, underscoring the significant impact a single adverse factor can have on implant prognosis. Notably, a substantial difference was observed between the questionable and unfavorable groups, with only 6.5% of failures in the questionable group, in contrast to the 66.7% in the unfavorable group. The high failure rate observed in the unfavorable group (HR = 9.70) highlights how the combination of active disease and influencing factors reduces the predictability of outcomes and limits prognosis certainty.

According to the findings of our study, uncontrolled diabetes plays an essential role in determining implant survivability. Diabetes mellitus is a metabolic disorder characterized by impaired blood glucose regulation due to defects in insulin secretion, insulin action, or both.^13^ Uncontrolled diabetes can lead to dysregulation of our immune response, causing an abnormal inhibition of neutrophil's bacteria killing capability while also increasing responsiveness of macrophages to bacterial antigens, the culmination of which leads to the amplification of periodontal destruction.^14^ Although the underlying molecular mechanisms and pathogenesis of diabetes have been robustly studied and are largely well‐understood, the World Workshop in 2017^15^ highlighted conflicting evidence in determining whether diabetes control is a risk factor/indicator for peri‐implantitis.^16^, ^17^, ^18^ The findings of our study align with a retrospective cohort study reporting an increased in implant failure rate in individuals with diabetes (risk ratio = 2.75).^19^ Similarly, a systematic review and meta‐analysis found that patients with diabetes had approximately a 50% higher risk of developing peri‐implantitis compared to non‐diabetic individuals (risk ratio = 1.46).^20^ Among non‐smokers, those with hyperglycemia were 3.39 times more likely to develop peri‐implantitis than those with well‐controlled blood glucose levels. Conversely, a 5‐year cohort study observed no significant association between diabetes and the progression of peri‐implantitis in individuals previously diagnosed with peri‐implant mucositis.^21^ It is important to note that many of these studies were limited by small sample sizes; thus, larger and more comprehensive studies are warranted to strengthen the evidence base.

Additionally, our study found a significant association between a history of periodontitis and implant prognosis. This finding is supported by a systematic review indicating that numerous longitudinal and cross‐sectional studies have identified a prior history of periodontitis as a key risk factor or indicator for peri‐implantitis.^15^ A retrospective study by Renvert et al. reported that individuals with a history of chronic periodontitis were 4.5 times more likely to develop peri‐implantitis compared to those without such a history.^22^ Several studies propose that pathogenic bacteria associated with periodontitis may serve as a microbial reservoir, capable of colonizing newly placed implants and thereby increasing susceptibility to peri‐implant disease.^23^ *^–^* ^25^ For instance, one study examined the microbiota surrounding healthy implants in individuals previously treated for aggressive periodontitis and identified low levels of *Porphyromonas gingivalis*, *Tannerella forsythia*, and *Treponema denticola*.^26^ These microorganisms, collectively known as the red complex, are strongly associated with both periodontal disease and peri‐implantitis.^27^ Even in low quantities, their presence may elevate a patient's risk for future peri‐implant complications.

In our study, individuals with a history of grade C periodontitis, characterized by rapid disease progression, exhibited impaired implant survivability. This aligns with a systematic review and meta‐analysis showing that individuals with aggressive periodontitis are more prone to implant loss compared to those with chronic periodontitis or a healthy periodontium.^28^ Specifically, patients with aggressive periodontitis were nearly four times more likely to experience implant failure than those with chronic periodontitis. Moreover, a long‐term prospective study spanning 3 to 16 years demonstrated that patients treated for generalized aggressive periodontitis had significantly higher risks of peri‐mucositis (three times), peri‐implantitis (14 times), and implant failure (five times) compared to periodontally healthy controls.^29^ It should be acknowledged that the large HR observed for grade C periodontitis should be interpreted with caution due to the limited number of failure events. Nevertheless, the overall trend supports an association between a history of grade C periodontitis and compromised implant survivability. Regarding the frequency of supportive care, the present study found no significant difference in the risk of implant loss between patients who received supportive care more than twice per year and those who received it once annually. This result contrasts with existing literature, which has consistently shown a strong association between supportive care frequency and the prevention of peri‐implant diseases. Previous studies have shown that supportive care and recall intervals are closely associated with developing peri‐implant diseases.^10^, ^30^, ^31^ In a cross‐sectional study, patient compliant with peri‐implant maintenance therapy (PIMT) was associated with an 86% lower incidence of peri‐implantitis, indicating that PIMT provided more than twice per year was critical in preventing diseases.^10^ In another 11‐year prospective study, a higher incidence of peri‐implant mucositis and peri‐implantitis was observed among patients with irregular compliance.^30^ The odd ratio of peri‐implant disease was 4.65 in irregularly compliant patients compared to 1.92 in regularly compliant patients. The implant survival rate was also 3.4% higher in the regular compliance group. This discrepancy may be attributed to the retrospective nature of the present study. The frequency of maintenance visits may not accurately represent true patient compliance, as more frequent appointments could reflect either increased disease susceptibility or greater motivation for preventive care. Those who returned for maintenance more regularly may have had a more aggressive form of the disease or additional risk indicators contributing to implant loss, thereby necessitating more frequent follow‐ups. Additionally, the retrospective nature of the study precluded assessment of patients' oral hygiene and the quality of supportive care received, which may have limited our ability to accurately evaluate the true impact of supportive care frequency. Therefore, a prospective study is warranted to investigate the relationship between maintenance frequency and implant outcomes.

In the implant prognosis system proposed by Kwok et al.,^9^ both systemic and local factors are considered, including plaque control, smoking history, supportive care, diabetes control, osteoporosis, the use of bone sparing agents, radiation therapy, occlusion, and genetic factors. While the significance of certain factors warrants further investigation,^15^, ^32^ several important variables were not accounted for in the current system. For instance, implant phenotype plays a critical role, particularly the presence of peri‐implant keratinized mucosa, which has been shown to reduce plaque accumulation and the risk of marginal bone loss.^33^, ^34^ Additionally, although prosthetic factors were considered as local retentive factors in the original prognosis system,^9^ the full scope of these factors, such as the presence of residual cement^35^, prosthesis emergence angle and profile^36^, abutment height^37^, type of prosthetic restoration^38^, were not fully explored. Moreover, our study demonstrated higher predictability in mandibular implants compared to maxillary implants, likely influenced by differences in bone quality and density,^39^ resulting in a higher survival rate for implants placed in the mandible. This finding may also serve as an important consideration for future prognosis systems. Furthermore, the use of antidepressants has been associated with an increased risk of dental implant failure at both the patient and implant levels.^40^, ^41^ Future studies are warranted to further investigate the influence of these variables on the accuracy of prognosis. Overall, the implant prognosis system proposed by Kwok et al. represents a dynamic and evolving model. As additional evidence emerges, further refinement and updates may be necessary to enhance its predictive utility.

In comparison to the implant prognosis system proposed by Kwok et al., the therapeutic prognosis framework developed by Decker et al.^8^ lacks consideration of several critical factors that may influence long‐term implant survival. Notably, patient‐related variables such as systemic health conditions, smoking history, and the use of bone sparing agents were not incorporated. While Decker et al.’s system does evaluate site‐related factors, including the extent of bone loss and peri‐implant tissue destruction, it assumes ideal implant placement prior to assigning a prognosis. This assumption overlooks the clinical reality that implants placed outside of the bony housing can result in suboptimal defect configurations, thereby compromising the outcomes of peri‐implant reconstructive therapy.^42^ In contrast, the system proposed by Kwok et al. explicitly accounts for implant positioning, identifying implants placed at inappropriate angles or in non‐restorable positions as having a hopeless prognosis. This inclusion enhances the clinical applicability and predictive accuracy of Kwok et al.’s model for assessing implant survival.

This study has several limitations. First, due to its retrospective design, certain variables such as occlusal factors, genetic phenotypes, and detailed information on daily cigarette consumption could not be evaluated, potentially limiting assessment of dose‐dependent effects of smoking. Secondly, self‐reported data may introduce recall and confirmation biases, potentially misrepresenting patient's health status. Third, only one patient in the cohort was identified as having received IV bone sparing agents, limiting the ability to assess the impact of IV bone sparing agents on implant prognosis. Additionally, adjustments for multiple implants per patient were not feasible in this analysis due to the very limited number of implant failures occurring within the same patient. A larger sample size would also enhance the evaluation of successful implant outcomes and allow more for precise identification of risk factors contributing to implant failures. Finally, the mean follow‐up duration was less than 5 years; thus, extending the observation period would be valuable for assessing the long‐term (> 5 years) predictive accuracy of the implant prognosis system proposed by Kwok et al. 2023.^9^

Another limitation to the present study was the overarching classification of grade C periodontitis. According to Kwok et al.^9^, the presence of grade C periodontitis‐formerly known as aggressive periodontitis‐ was identified as a significant influencing factor associated with a heightened risk of implant loss. However, under the 2017 World Workshop classification system,^43^ grade C encompasses a broader range of grade modifiers beyond those defined under the 1999 classification of aggressive classification.^44^ In our study cohort, many patients were affected by uncontrolled diabetes and/or heavy cigarette smoking, both of which are recognized modifiers contributing to a grade C diagnosis. This heterogeneity suggests that the interaction between specific grade C modifiers and implant prognosis warrants further investigation to better understand their impact on disease progression and implant survival.

## 5 CONCLUSIONS

The dental implant prognosis system proposed by Kwok et al. in 2023 offers a reliable tool for assessing implant survivability within a 5‐year period. Future research should investigate its long‐term performance and the interactions between various influencing factors and implant prognosis.

## AUTHOR CONTRIBUTIONS

**Kristen Young**: Data curation; formal analysis; visualization; writing—original draft. **Michelle Tong**: Data curation; formal analysis. **Yu‐Ting Yeh**: Validation; writing—original draft preparation. **Richard T. Kao and Guo‐Hao Lin**: Conceptualization; methodology; project administration; writing—review & editing.

## CONFLICT OF INTEREST STATEMENT

The authors declare no conflicts of interest.

## Supporting information



Supporting Information

## Data Availability

The data sets used and/or analyzed during the current study are available from the corresponding author upon reasonable request.

## References

[1] Hirschfeld L , Wasserman B . A long‐term survey of tooth loss in 600 treated periodontal patients. J Periodontol. 1978;49:225‐237. 277674 10.1902/jop.1978.49.5.225

[2] Becker W , Berg L , Becker BE . The long term evaluation of periodontal treatment and maintenance in 95 patients. Int J Periodontics Restorative Dent. 1984;4:54‐71. 6589217

[3] McGuire MK . Prognosis versus actual outcome: a long‐term survey of 100 treated periodontal patients under maintenance care. J Periodontol. 1991;62:51‐58. 2002432 10.1902/jop.1991.62.1.51

[4] Kwok V , Caton JG . Commentary: prognosis revisited: a system for assigning periodontal prognosis. J Periodontol. 2007;78:2063‐2071. 17970671 10.1902/jop.2007.070210

[5] Branemark PI , Adell R , Breine U , Hansson BO , Lindstrom J , Ohlsson A . Intra‐osseous anchorage of dental prostheses. I. Experimental studies. Scand J Plast Reconstr Surg. 1969;3:81‐100. 4924041 10.3109/02844316909036699

[6] Elani HW , Starr JR , Da Silva JD , Gallucci GO . Trends in dental implant use in the U.S., 1999‐2016, and projections to 2026. J Dent Res. 2018;97:1424‐1430. 30075090 10.1177/0022034518792567 PMC6854267

[7] Atieh MA , Alsabeeha NH , Faggion CM Jr , Duncan WJ . The frequency of peri‐implant diseases: a systematic review and meta‐analysis. J Periodontol. 2013;84:1586‐1598. 23237585 10.1902/jop.2012.120592

[8] Decker AM , Sheridan R , Lin GH , Sutthiboonyapan P , Carroll W , Wang HL . A prognosis system for periimplant diseases. Implant Dent. 2015;24:416‐421. 26035268 10.1097/ID.0000000000000276

[9] Kwok V , Caton JG , Hart ID , Kim TS . Dental implant prognostication: a commentary. J Periodontol. 2023;94:713‐721. 36740787 10.1002/JPER.22-0196

[10] Monje A , Wang HL , Nart J . Association of preventive maintenance therapy compliance and peri‐implant diseases: a cross‐sectional study. J Periodontol. 2017;88:1030‐1041. 28548886 10.1902/jop.2017.170135

[11] von Elm E , Altman DG , Egger M , et al. The Strengthening the Reporting of Observational Studies in Epidemiology (STROBE) statement: guidelines for reporting observational studies. Int J Surg. 2014;12:1495‐1499. 25046131 10.1016/j.ijsu.2014.07.013

[12] Nguyen L , Krish G , Alsaleh A , et al. Analyzing the predictability of the Kwok and Caton periodontal prognosis system: a retrospective study. J Periodontol. 2021;92:662‐669. 33011996 10.1002/JPER.20-0411

[13] American Diabetes Association . Diagnosis and classification of diabetes mellitus. Diabetes Care 2010;33 Suppl 1:S62‐69. 20042775 10.2337/dc10-S062 PMC2797383

[14] Mealey BL , Oates TW , American Academy of Periodontology Commisioned Review: Diabetes mellitus and periodontal diseases. J Periodontol. 2006;77:1289‐1303. 16881798 10.1902/jop.2006.050459

[15] Schwarz F , Derks J , Monje A , Wang HL . Peri‐implantitis. J Clin Periodontol. 2018;45 (Suppl 20):S246‐S266. 29926484 10.1111/jcpe.12954

[16] Dalago HR , Schuldt Filho G , Rodrigues MA , Renvert S , Bianchini MA . Risk indicators for peri‐implantitis. A cross‐sectional study with 916 implants. Clin Oral Implants Res. 2017;28:144‐150. 26754342 10.1111/clr.12772

[17] Derks J , Schaller D , Hakansson J , Wennstrom JL , Tomasi C , Berglundh T . Effectiveness of implant therapy analyzed in a Swedish population: prevalence of peri‐implantitis. J Dent Res. 2016;95:43‐49. 26701919 10.1177/0022034515608832

[18] Rokn A , Aslroosta H , Akbari S , Najafi H , Zayeri F , Hashemi K . Prevalence of peri‐implantitis in patients not participating in well‐designed supportive periodontal treatments: a cross‐sectional study. Clin Oral Implants Res. 2017;28:314‐319. 26919480 10.1111/clr.12800

[19] Moy PK , Medina D , Shetty V , Aghaloo TL . Dental implant failure rates and associated risk factors. Int J Oral Maxillofac Implants. 2005;20:569‐577. 16161741

[20] Monje A , Catena A , Borgnakke WS . Association between diabetes mellitus/hyperglycaemia and peri‐implant diseases: systematic review and meta‐analysis. J Clin Periodontol. 2017;44:636‐648. 28346753 10.1111/jcpe.12724

[21] Costa FO , Takenaka‐Martinez S , Cota LO , Ferreira SD , Silva GL , Costa JE . Peri‐implant disease in subjects with and without preventive maintenance: a 5‐year follow‐up. J Clin Periodontol. 2012;39:173‐181. 22111654 10.1111/j.1600-051X.2011.01819.x

[22] Renvert S , Aghazadeh A , Hallstrom H , Persson GR . Factors related to peri‐implantitis—a retrospective study. Clin Oral Implants Res. 2014;25:522‐529. 23772670 10.1111/clr.12208

[23] Canullo L , Penarrocha‐Oltra D , Covani U , Botticelli D , Serino G , Penarrocha M . Clinical and microbiological findings in patients with peri‐implantitis: a cross‐sectional study. Clin Oral Implants Res. 2016;27:376‐382. 25622536 10.1111/clr.12557

[24] Mombelli A , Marxer M , Gaberthuel T , Grunder U , Lang NP , The microbiota of osseointegrated implants in patients with a history of periodontal disease. J Clin Periodontol 1995;22:124‐130. 7775668 10.1111/j.1600-051x.1995.tb00123.x

[25] Quirynen M , Listgarten MA . Distribution of bacterial morphotypes around natural teeth and titanium implants ad modum Branemark. Clin Oral Implants Res 1990;1:8‐12. 2099212 10.1034/j.1600-0501.1990.010102.x

[26] De Boever AL , De Boever JA . Early colonization of non‐submerged dental implants in patients with a history of advanced aggressive periodontitis. Clin Oral Implants Res 2006;17:8‐17. 10.1111/j.1600-0501.2005.01175.x 16441780

[27] Di Spirito F , Giordano F , Di Palo MP , et al. Microbiota of peri‐implant healthy tissues, peri‐implant mucositis, and peri‐implantitis: a comprehensive review. Microorganisms 2024;12(6):1137. 38930519 10.3390/microorganisms12061137 PMC11205430

[28] Monje A , Alcoforado G , Padial‐Molina M , Suarez F , Lin GH , Wang HL . Generalized aggressive periodontitis as a risk factor for dental implant failure: a systematic review and meta‐analysis. J Periodontol 2014;85:1398‐1407. 24835415 10.1902/jop.2014.140135

[29] Swierkot K , Lottholz P , Flores‐de‐Jacoby L , Mengel R . Mucositis, peri‐implantitis, implant success, and survival of implants in patients with treated generalized aggressive periodontitis: 3‐ to 16‐year results of a prospective long‐term cohort study. J Periodontol 2012;83:1213‐1225. 22264211 10.1902/jop.2012.110603

[30] Costa FO , Costa AM , Ferreira SD , et al. Long‐term impact of patients' compliance to peri‐implant maintenance therapy on the incidence of peri‐implant diseases: an 11‐year prospective follow‐up clinical study. Clin Implant Dent Relat Res 2023;25:303‐312. 36519351 10.1111/cid.13169

[31] Pjetursson BE , Helbling C , Weber HP , et al. Peri‐implantitis susceptibility as it relates to periodontal therapy and supportive care. Clin Oral Implants Res 2012;23:888‐894. 22530771 10.1111/j.1600-0501.2012.02474.x

[32] Curtis DA , Lin GH , Fishman A , et al. Patient‐centered risk assessment in implant treatment planning. Int J Oral Maxillofac Implants 2019;34:506‐520. 30716143 10.11607/jomi.7025

[33] Mancini L , Strauss FJ , Lim HC , et al. Impact of keratinized mucosa on implant‐health related parameters: a 10‐year prospective re‐analysis study. Clin Implant Dent Relat Res 2024;26:554‐563. 38419210 10.1111/cid.13314

[34] Roccuzzo M , Grasso G , Dalmasso P . Keratinized mucosa around implants in partially edentulous posterior mandible: 10‐year results of a prospective comparative study. Clin Oral Implants Res 2016;27:491‐496. 25706508 10.1111/clr.12563

[35] Kordbacheh Changi K , Finkelstein J , Papapanou PN . Peri‐implantitis prevalence, incidence rate, and risk factors: a study of electronic health records at a U.S. dental school. Clin Oral Implants Res 2019;30:306‐314. 30768875 10.1111/clr.13416

[36] Katafuchi M , Weinstein BF , Leroux BG , Chen YW , Daubert DM . Restoration contour is a risk indicator for peri‐implantitis: a cross‐sectional radiographic analysis. J Clin Periodontol 2018;45:225‐232. 28985447 10.1111/jcpe.12829

[37] Spinato S , Stacchi C , Lombardi T , Bernardello F , Messina M , Zaffe D . Biological width establishment around dental implants is influenced by abutment height irrespective of vertical mucosal thickness: a cluster randomized controlled trial. Clin Oral Implants Res 2019;30:649‐659. 31033035 10.1111/clr.13450

[38] AlRowis R , Albelaihi F , Alquraini H , Almojel S , Alsudais A , Alaqeely R . Factors affecting dental implant failure: a retrospective analysis. Healthcare (Basel) 2025;13(12):1356. 40565383 10.3390/healthcare13121356 PMC12193482

[39] Becker W , Becker BE , Alsuwyed A , Al‐Mubarak S . Long‐term evaluation of 282 implants in maxillary and mandibular molar positions: a prospective study. J Periodontol 1999;70:896‐901. 10476898 10.1902/jop.1999.70.8.896

[40] Carr AB , Gonzalez RLV , Jia L , Lohse CM . Relationship between selective serotonin reuptake inhibitors and risk of dental implant failure. J Prosthodont 2019;28:252‐257. 30637850 10.1111/jopr.13015

[41] Harutyunyan L , Lieuw K , Yang B , et al. The effect of antidepressants on dental implant failure: a systematic review and meta‐analysis. Int J Oral Maxillofac Implants 2024;39:665‐673. 38498803 10.11607/jomi.10798

[42] Monje A , Pons R , Insua A , Nart J , Wang HL , Schwarz F . Morphology and severity of peri‐implantitis bone defects. Clin Implant Dent Relat Res 2019;21:635‐643. 31087457 10.1111/cid.12791

[43] Papapanou PN , Sanz M , Buduneli N , et al. Periodontitis: consensus report of workgroup 2 of the 2017 World Workshop on the Classification of Periodontal and Peri‐Implant Diseases and Conditions. J Periodontol 2018;89 Suppl 1:S173‐S182. 29926951 10.1002/JPER.17-0721

[44] Armitage GC . Development of a classification system for periodontal diseases and conditions. Ann Periodontol 1999;4:1‐6. 10863370 10.1902/annals.1999.4.1.1

