# Supplementary material for: Predictability of a dental implant prognosis system: A retrospective study
Source: J Periodontol. 2026 Mar 6;97(8):1672–82. doi: 10.1002/jper.70110 (PMC13502445; doi:10.1002/jper.70110)
Supplement: Supplementary file 1 [file JPER-97-1672-s001.docx]

**Supplemental Table 1:** Prognosis category based on determining factors that can affect the stability of peri-implant supporting tissue.

| **Prognosis** | **Criteria** | **Outcome** |
| --- | --- | --- |
| Favorable | No peri-implantitis (peri-mucositis allowed); no influencing factors present | Stability likely to be maintained |
| Questionable | Peri-implantitis or significant negative influencing factors present, but not both | Stability may not be maintained |
| Unfavorable | Peri-implantitis and influencing factors present | Stability unlikely to be maintained |
| Hopeless | Lack of osseointegration, severe malposition, or a non-restorable implant requiring submergence | Implant cannot be maintained |
